# Unusual MRI findings in an immunocompetent patient with EBV encephalitis: a case report

**DOI:** 10.1186/1471-2342-11-6

**Published:** 2011-03-24

**Authors:** Paola Di Carlo, Marcello Trizzino, Lucina Titone, Giuseppina Capra, Piero Colletti, Giovanni Mazzola, Daniela Pistoia, Caterina Sarno

**Affiliations:** 1 Dipartimento di Scienze per la Promozione della Salute, Università di Palermo, Italy; 2 Dipartimento di Medicina Clinica e delle Patologie Emergenti, Azienda Ospedaliera Universitaria Policlinico "Paolo Giaccone" di Palermo, Palermo, Italy; 3 Dipartimento di Scienze Radiologiche, Azienda Ospedaliera Universitaria Policlinico "Paolo Giaccone" di Palermo, Palermo, Italy

**Keywords:** Epstein-Barr virus, encephalitis, MRI, Diffusion-weighted imaging

## Abstract

**Blackground:**

It is well-known that Epstein-Barr virus (EBV) can affect the central nervous system (CNS).

**Case presentation:**

Herein the authors report unusual timely Magnetic Resonance Imaging (MRI) brain scan findings in an immunocompetent patient with EBV encephalitis.

Diffusion weighted MRI sequence performed during the acute phase of the disease was normal, whereas the Fast Relaxation Fast Spin Echo T2 image showed diffuse signal intensity changes in white matter. The enhancement pattern suggested an inflammatory response restricted to the brain microcirculation. Acyclovir and corticosteroid therapy was administered. After three weeks, all signal intensities returned to normal and the patient showed clinical recovery.

**Conclusion:**

This report demonstrates that EBV in an immunocompetent adult can present with diffuse, reversible brain white matter involvement in the acute phase of mononucleosis. Moreover, our case suggests that a negative DWI sequence is associated with a favorable improvement in severe EBV CNS infection. More extensive studies are needed to assess what other instrumental data can help to distinguish viral lesions from other causes in the acute phase of disease.

## Background

Epstein-Barr virus (EBV) is the causative agent of infectious mononucleosis (IM) and can lead to various central nervous system (CNS) complications, such as demyelinating disease, acute encephalitis, meningitis and acute cerebellar ataxia [1-3]. The illness usually runs a benign course, though fatal cases have been reported [4].

Neurological involvement has been reported in children during the acute phase of the disease and sequelae occur in a substantial number of patients [4-6]. Neurological manifestations associated with reactivation of EBV infection in immunocompetent adults have recently been reported [7,8]. Furthermore, evidence suggests that EBV plays a role in multiple sclerosis (MS) disease activity and in Acute disseminate encephalomyelitis (ADEM), an immune-mediated inflammatory disorder of the CNS, characterized by a multifocal demyelination that involves the white matter of the brain and spinal cord [9,10].

Magnetic resonance imaging (MRI) shows small or multiple CNS lesions more clearly, allowing rapid diagnosis and the formulation of more effective therapeutic strategies [6,11]. Diffusion-weighted imaging (DWI) sequence identifies CNS lesions earlier than T2W or Fluid Attenuated Inversion Recovery (FLAIR) imaging [11-15].

Herein we report an unusual timely CNS imaging sequence in an immunocompetent patient with acute infectious mononucleosis.

## Case presentation

A 19-year-old Italian male student came to the Emergency Department of the "Policlinico Paolo Giaccone" University Hospital in Palermo, Italy, with fever, headache and altered consciousness. The fever started one week before presentation; he had experienced pharyngitis, lymphadenopathy and asthenia. He had no other history of disease and was immunocompetent. An initial cranial CT scan was normal.

On admission, he had a Glasgow score of 12 and a temperature of 38.6°C; he had visual disorders including blurred vision, impaired accommodation and diplopia.

He presented with cervical lymphadenopathy and hepatosplenomegaly.

Laboratory tests revealed that he had a white blood cell count of 15400 cell mm^-3^, with 20% neutrophils, 36,2% lymphocytes, 33,1% monocytes and hypertransaminasemia (aspartate aminotransferase level of 78 IU l^-1 ^and an alanine aminotransferase level of 271 IU l^-1^).

Lumbar puncture revealed 81 cells/μL with 27% mononuclear cells and a protein content of 102 mg dl^-1^.

Real Time quantitative PCR of Cerebrospinal Fluid (CSF) was positive for EBV (2.200 copies/mL). We used the BAMHI-W fragment region of the EBV genome as the target of our PCR screening [16].

PCR of the CSF sample was negative for Herpes Virus 1 And 2, Varicella Zoster Virus, Cytomegalovirus and Enterovirus. Bacterial cultures were negative for *Mycobacterium tuberculosis *and other pathogens which are epidemiologically relevant in our geographic area (e.i. *Rickettsia conorii, Brucella spp*).

On admission, serological tests for HIV, VDRL, CMV, HSV, hepatitis A, hepatitis C, b Borrelia and Brucella were all negative. Serological tests for EBV confirmed primary infection: VCA IgM positive, EA IgG positive, VCA IgG positive, and EBNA IgG negative.

An MRI scan of the brain using gadolinium contrast enhancement was performed 18 hours after the patient was admitted to hospital. Images were taken of the axial, coronal and sagittal sections.

MRI was initially performed with a 1.5-T unit (General Electric, United State) with a single channel receiver coil and body coil for excitation. The examination protocol included FRFSE T2W, FLAIR T2W, DWI and FSE T1W before and after intravenous administration of Gadobutrol. A power injector was used to administer 0,1 mL of Gadovist^® ^(Gadobutrol, 1 M, Bayer Schering Pharma AG, Berlin, Germany) per kg of body weight (equivalent to 0,1 mmol/kg body weight) followed by 20 ml of saline. Injection rate was 2 mL/s. Sequence parameters are reported in Table 1.

**Table 1:** MRI sequence parameters.

	T2WI	DWI	FLAIR	T1WI
*TR, ms*	4325	7000	9000	500
*TE, ms*	102	98	118	10,8
*b, s/mm*^2^	-	1000	-	-
*FOV, mm*	220	200	220	220
*Matrix*	256 × 256	128 × 128	256 × 256	256 × 256
*Thickness, mm*	5,0	5,0	5,0	5,0
*Slices, n*	22	44	22	22

**Figure 1:**
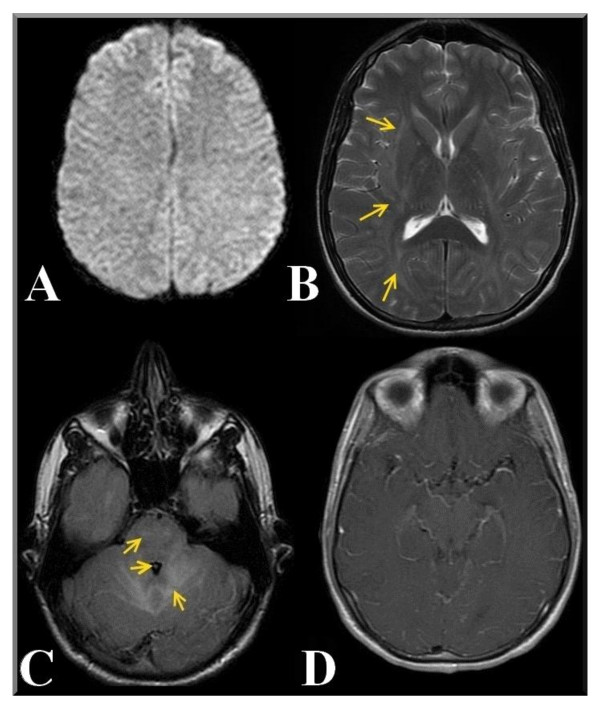
**Initial MR imaging of the patient**. Magnetic Resonance Imaging of the brain in the acute phase of neurological involvement. Diffusion Weighted MR Imaging of the brain performed in the acute phase of encephalitis (A). Axial T2 FRFSE image shows hyperintensity of white matter with involvement of internal and external capsule (B). Axial T2 FLAIR image demonstrates hyperintensity of white matter; involvement of the ventricular system with reduction in the size of the fourth ventricle is also shown (C). On the T1W Axial FLAIR there is enhancement of cerebral vessels after contrast agent was administered. Hyperintensity of cerebral vessel (D).

The DWI sequence did not show any signal abnormalities (figure 1, A) whereas the T2 Fast relaxation fast spin echo sequence detected demyelinating alteration of the sub- and supra-tentorial white matter, and internal and external capsule and periventricular white matter and semioval center involvement, with scattered, partly confluent white matter changes (figures 1, B)

Fourth ventricle size was reduced, which is consistent with cerebral edema, and we also observed hyperintensity in the ponto-mesencephalic region (cortico spinal tract), on the T2 FLAIR sequence. (figure 1, C).

Contrast agent enhancement pattern suggested an inflammatory response restricted to the brain microcirculation (figure 1, D).

The patient was treated with intravenous acyclovir (10 mg/kg every 8 h) combined with methylprednisolone (500 mg intravenous bolus for 3 days then 1 mg/kg daily for 21 days).

Neurological improvement was noted at day 10 (Glasgow score of 14) but his diplopia symptoms persisted. PCR on CSF performed three weeks after admission was negative for EBV.

**Figure 2:**
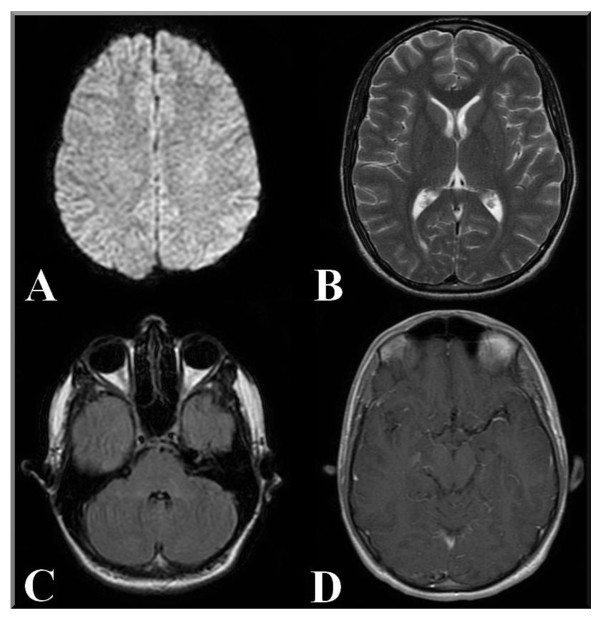
**MR imaging of the patient at follow-up 3 weeks later**. DWI remains the same, without any signal alteration (A). On the Axial T2 FRFSE image, we can see substantial regression of the previous lesions and no new lesions (B). The Axial T2 FLAIR image shows that the fourth ventricle has returned to its normal size. All signal intensities are normalized (C). On the T1W Axial FLAIR there is no enhancement of cerebral vessels after contrast medium was administered, as described above (D).

An MRI scan of the brain after three weeks showed markedly decreased hyperintensity on T2W and contrast enhancement had disappeared on post-gadolinium T1-W (figure 2, B,C,D).

Scanner and sequence parameters were identical to those used previously (Table 1).

Six months later, the patient had residual diplopia but was otherwise normal.

## Discussion

A brain infection is a medical emergency. Immediate diagnosis and initiation of symptomatic and specific therapy has a dramatic influence upon survival and reduces the extent of permanent brain damage in survivors. In cases of herpes virus encephalitis, EBV has rarely been reported as the cause of CNS lesions, although fatal events and severe sequelae have been described, mostly in cases of acute primary infection [17,18].

DWI sequences provide image contrast that is dependent on the molecular motion of water; this is important in cases of increased or restricted diffusion, such as ischemic stroke, intracranial infections or trauma[6,11-13]. Viral infections of the nervous system are characterized by alterations in water diffusion caused by cytotoxic edema, which can be detected by DWI. Reports have shown that DWI is usually the first sequence that is activated during cytotoxic cortical edema in tissue undergoing necrosis in EBV encephalitis [11-15].

In our case, we did not detect any abnormal signs in the DWI sequence, whereas T2W images showed mainly sub- and supra-tentorial white matter demyelination.

Our young patient's favorable course of CNS EBV infection and clearance suggests that EBV damage may be reversible when no abnormalities are detected on DWI, and appropriate therapy is initiated [14].

Apparent Diffusion Coefficient (ADC) maps can predict outcome in acute encephalitis [15]; we did not calculate this at the time of admission as this feature is not available at our hospital under emergency conditions.

We observed diffuse cerebral micro circular enhancement on T1W images after contrast agent was administered. In the course of inflammatory neurological disorders, adhesion molecule expression on endothelial cells is increased. This amplifies the inflammatory process in viral encephalitis where there is a lymphocytic infiltration of the CNS [19]. Research studies conducted on animals suggest that the herpes virus has an endotheliotropism that may depend on the susceptibility of CNS microvascular endothelial cells [20]. Moreover, EBV infection of human brain microvessel endothelial cells has recently been associated with the onset of multiple sclerosis [21].

Besides other infectious agents are responsible for mononucleosis-like illnesses, such as cytomegalovirus, HIV, cat scratch disease and syphilis, we investigated additional pathogens which are responsible for meningo-encephalitis in our geographic area, such as tuberculosis, rickettsial and brucellosis diseases.

ADEM was one of the non-infective CNS inflammatory diseases which we considered in the differential diagnosis of our patient. ADEM is a monophasic autoimmune demyelinating disease of the CNS that may occur after an infection or vaccination. However, ADEM lesions involve basal ganglia, thalami and the brainstem in particular [10] and this was not the case for our patient. Furthermore, clinical and instrumental resolution does not occur as early as it did in this case.

EBV-specific antiviral therapy does not exist, but acyclovir or gancyclovir have been recommended for the treatment of CNS involvement [6,7]. There is some debate surrounding corticosteroid therapy, but it seems to be a reasonable option when vasculitis is suspected, as our patient's neuroimaging findings suggested.

In conclusion, this report demonstrates that EBV in an immunocompetent adult can present with diffuse, reversible brain white matter involvement in the acute phase of mononucleosis.

## Consent

Written informed consent was obtained from the patient for publication of this case report and any accompanying images.

## List of abbreviations

ADC: Apparent Diffusion Coefficient; ADEM: Acute Disseminated Encephalomyelitis; CNS: Central Nervous System; CSF: Cerebrospinal Fluid; CT: Computed Tomography; DWI: Diffusion Weighted Imaging; EA: Early Antigen; Ig: Immunoglobulin; EBNA: Epstein-Barr virus nuclear antigen; EBV: Epstein-Barr Virus; FLAIR: Fluid Attenuated Inversion Recovery; FRFSE: Fast Relaxation Fast Spin Echo; FSE: Fast Spin Echo; MRI: Magnetic Resonance Imaging; PCR: Polymerase Chain Reaction; T1W: T1-weighted; T2W: T2-weighted; VCA: Viral Capsid Antigen; VDRL: Venereal Disease Research Laboratory test; CMV: Cytomegalovirus; HIV: Human immunodeficiency virus; HSV: Herpes Simplex Virus.

## Competing interests

The authors declare that they have no competing interests.

## Authors' contributions

PD, MT and CS developed the idea of the study, participated in its design and coordination and helped to draft the manuscript. PC, GM and LT contributed to the acquisition and interpretation of data. DP and GC performed and collected laboratory tests and molecular genetic studies. CS was involved in critically reviewing neuro-imaging data for important intellectual content. All authors read and approved the final manuscript.

## Pre-publication history

The pre-publication history for this paper can be accessed here:

http://www.biomedcentral.com/1471-2342/11/6/prepub
